# Interacting effects of density and temperature on fish growth rates in freshwater protected populations

**DOI:** 10.1098/rspb.2021.1982

**Published:** 2022-01-26

**Authors:** Andrew S. Watson, Michael J. H. Hickford, David R. Schiel

**Affiliations:** Marine Ecology Research Group, School of Biological Sciences, University of Canterbury, Private Bag 4800, Christchurch 8140, New Zealand

**Keywords:** fisheries management, freshwater protected areas, *Galaxias maculatus*, īnanga, New Zealand, whitebait

## Abstract

Despite the demonstrated benefits of marine protected areas, there has been relatively little dialogue about freshwater protected areas (FPAs) even though some have been established to protect freshwater species from recreational and commercial fishers. After populations recover from fishing pressure, abundances and densities of formerly fished species increase, and we should therefore expect changes in demographic traits compared to those in exploited populations. To test this, we used capture-mark-recapture data for 10 *Galaxias maculatus* populations across a density gradient mediated by different degrees of fishery closure. We examined the extent to which density-dependent (DD) and density-independent (DI) effects interact to affect specific growth rates in post-recruit populations. We found that population density, stream temperature and individual size interact to affect growth rates. When population densities were high, compensatory responses of far slower growth rates were strongest, indicating that DD growth is a key mechanism regulating post-recruit populations of *G. maculatus*. This study emphasizes the importance of understanding DD and DI processes, their interactions, function and effectiveness for freshwater fisheries management. For FPAs to be effective, the extent and quality of target species' habitats must serve as key criteria for protection to alleviate competition for limited resources that underpins DD processes.

## 1. Introduction

The last century has seen an expansion of protected areas (PAs) worldwide [1]. Marine protected areas (MPAs), in particular, have been embraced as management tools for biodiversity conservation and fisheries enhancement. Despite the demonstrated benefits of MPAs, there has been little dialogue and analyses around the benefits of freshwater protected areas (FPAs). Although relatively rare, some FPAs have been established to protect freshwater species and habitats [2]. The most common of these are Ramsar sites, which are often classified as PAs even though many are managed by non-governmental managers or without specific legislation [3]. As of 2021, the Ramsar List contained 2433 sites covering over 2.5 million km^2^, which represents about 20% of the estimated 12.8 million km^2^ of global wetlands [4]. Besides Ramsar sites, few other FPAs exist [3]. However, with 71% of freshwater fish extinctions being attributed in part to habitat loss and with some species at risk from overharvesting [5], FPAs have never been more topical.

The reserve effects that typically occur after the establishment of MPAs, when compared with unprotected areas, include increased average ages and sizes of individuals within fish populations, increased abundances and densities, enhanced reproductive output, increased biodiversity, improved habitat complexity and shifts in ecosystem function [6,7]. These types of responses to protection are expected, but subsequent biological and ecological processes must be considered and understood to maximize the efficacy of PAs. For example, density-dependent (DD) changes in individual life-history characteristics should be expected when abundances and densities increase as a result of cessation or reduction of fishery harvest [8].

In general, the processes capable of determining population abundances can be classified as either DD or density-independent (DI), and determining the extent to which each of these regulates natural populations has long been a central theme in animal ecology [9]. Density dependence is a fundamental concept in the study of fish population dynamics, and the theory of compensatory density dependence underlies fisheries management [10]. Overall, processes such as growth, reproduction, survival and dispersal are ‘compensatory' if they slow population growth at high densities or increase population growth at low densities [10]. These processes are DI if their rates change in response to stochastic environmental factors such as temperature [10] and stream flow [11]. While the concept of compensatory density dependence is straightforward, it remains one of the most elusive issues in population dynamics because it requires an understanding of how DD and DI processes interact [10]. Historically, the debate over population regulation focused on the relative occurrence of either DD or DI processes [12], but it is now generally accepted that DD and DI processes act in concert [13] and that the strength of their interactions can vary spatio-temporally and by the demographic trait in question [14].

Although several studies have addressed DD responses in the context of MPAs [8], the link between DD growth and the effect of FPAs has not been well-explored. Here, we focused on understanding the relative strength of DD versus DI factors on individual growth rates in post-recruit populations of an annual amphidromous fish, *Galaxias maculatus* (locally called ı̄nanga). Understanding the links between environmental conditions and instantaneous growth can provide fundamental information about what limits the productivity of fish populations [14]. Our attempt to understand the interacting effects of DD and DI factors on *Galaxias maculatus* is the first we know of, and could therefore provide valuable information needed for fisheries conservation and management. This is particularly relevant because of threats to this species and many freshwater fishes worldwide, which include the loss of rearing, feeding and spawning habitats through riparian zone clearance, wetland drainage, loss of connectivity through river modifications, and predation and competition from introduced species.

Instantaneous growth is an essential component of fisheries assessments because body size correlates with many life-history attributes [14,15] and has been linked to foraging success [16], competitive ability [17], reproductive success [18] and size-selective mortality [19]. Ultimately, changes in growth rates at the individual level are translated upward to the population level and can result indirectly in reduced abundance, thus regulating populations [20]. Therefore, it is important to understand the extent to which DD and DI factors are manifested at the individual level and what they tell us about species' trait lability to underpin targeted conservation and fisheries management goals.

*Galaxias maculatus* is an annual, amphidromous species, and comprises up to 88% of New Zealand's post-larval ‘whitebait' catch [21]. During migration into coastal waterways, millions of post-larval *G. maculatus* run a gauntlet of fishers during the open season, as well as predatory fishes and birds [22]. Compared to other galaxiids, *G. maculatus* are bound to coastal watersheds and have poor climbing ability, which limits them to areas below waterfalls that other galaxiid species can ascend [23]. Adult *G. maculatus* are opportunistic feeders, taking a variety of terrestrial and aquatic organisms, and their diet varies greatly with habitat [24]. Adults inhabit a variety of habitats [23], and they spend their entire adult lives in the stream they enter as post-larvae [25]. Because of these spatial restrictions, and the vast abundance of returning post-larvae, there is a good case to hypothesise that DD processes strongly affect *G. maculatus* populations in their freshwater habitats, and that removal of large numbers of post-larvae through fishing may modify the strength of these processes.

In this study, we examined the additive and interactive effects of individual size, population density, benthic biomass, canopy closure, stream temperature and stream discharge on specific individual growth rates of *G. maculatus* across a density gradient mediated by areas closed to fishing [26]. We hypothesize that: (a) individual growth rates would decrease as population density increased due to DD effects, similar to those found in other temperate stream-dwelling fish [27]; (b) individual growth rates would increase with stream temperature because temperature is an important variable affecting development rates in ectotherms [15]; (c) individual growth rates would increase with increasing stream discharge due to increased availability of invertebrate drift [28]; (d) individual growth rates would decrease with benthic biomass and canopy closure due to reduced availability of terrestrial and stream-based prey; (e) DD effects would affect smaller individuals more strongly due to gape size limitation regulating their consumption of available prey; and (f) DD effects would interact with DI processes and exacerbate negative effects on individual growth. These effects are likely to be manifested and modified through the sizes of individuals, remaining numbers in populations, food availability, food quality and environmental conditions (e.g. temperature and stream flow). We chose to monitor post-recruit (late juvenile and adult) demography because larval and post-larval stages of *G. maculatus* occur at sea, are largely unknown and challenging to monitor. Furthermore, post-recruit demography may inherently reflect the demography of early life stages [29] and consequently their survival to adulthood.

## 2. Methods

### (a) *Galaxias maculatus* as a test species

*Galaxias maculatus* is a good study organism for testing DD and DI effects because it is abundant and shows high fidelity to individual streams and rivers, with little or no movement between them after they settle as post-larvae [25]. The species is the most commonly occurring species in the culturally and economically important ‘whitebait' fishery in New Zealand, which is focused on post-larval fishes as they migrate into freshwater from the sea [21]. Other whitebait fisheries based on migratory galaxiids also occur in Australia and South America, but on a much smaller scale than in New Zealand. There is anecdotal evidence suggesting the whitebait fishery is declining because of variable catches among years, but large annual fluctuations in harvest are typical [22], thereby confounding any longer term signal from which a decline could be determined. These irregular and frequent fluctuations are not consistent with typical observed declines in fish stocks characteristic of an overexploited fishery, and may imply some degree of long-term stability or high environmentally driven productivity around a longer-term more stable mean. The putative risks of overfishing were recognized long ago and as a precautionary conservation measure several areas were closed to whitebait fishing from the mid-1960s onwards [30]. Nevertheless, while a lack of definitive evidence of how populations are regulated remains, the debate over the long-term consequences of harvesting, habitat loss, and introduced species will continue. It is evident that many of the world's freshwater fisheries are deteriorating [31], and New Zealand's whitebait fishery is probably no exception.

### (b) Population study areas and sampling

**Figure 1.**
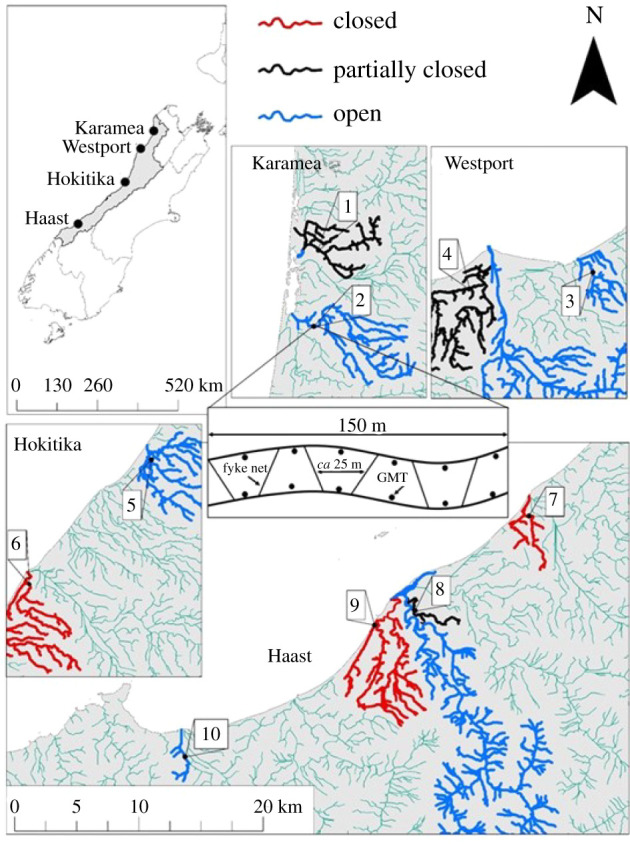
Map of the West Coast of New Zealand and the ten study sites sampled for īnanga (*Galaxias maculatus*) populations. Inset schematic shows how the six fyke nets and 12 Gee minnow traps (GMT) were spread throughout each site. (Online version in colour.)

We compared different FPAs, established to prevent fisheries exploitation, with unprotected, fished, areas along the West Coast of New Zealand. In total, 10 streams were included in this study (figure 1; electronic supplementary material, table S1). Of these, three streams were completely closed to whitebait fishing from the mouth to the upper catchment. We have called these no-take FPAs ‘closed'. Another three streams were tributaries closed to fishing but where whitebait could still be caught in the lower reaches below the closed tributary. We have called these partially protected freshwater areas ‘partially closed'. For comparison, four fished streams were selected based on catchment size, stream order and accessibility relative to the FPAs, and geographically interspersed among the two FPA types. We have called these unprotected areas ‘open'. Pervious work by [26] showed that on average, closed streams had around 10 times more juvenile *G. maculatus* than partially closed and open streams.

*Galaxias maculatus* populations were sampled monthly in set areas of streams delineated in 150 m transects (sites), located well upstream of spawning areas and fishing pressure (i.e. above tidally inundated riparian vegetation in coastal waterways). Sampling was done post-peak-migration from January to April 2019, and January to March 2020 (sampling was cut short by a month in 2020 due to COVID-19-related travel restrictions). At each site, *G. maculatus* were collected using standard trapping methods for lowland streams [32]. Captured fish were held in buckets of aerated stream water, anaesthetized with 0.5 ml l^−1^ of 2-phenoxyethanol then wet-weighed (±0.01 g) and the total length (TL) measured (±1 mm). During the first capture event of each year at each site, post-recruit *G. maculatus* were tagged with visible implant elastomer (Northwest Marine Technology, WA, USA). Tags consisted of unique combinations of three dye colours and six possible positions on the dorsal surface. During subsequent sampling, all captured fish were searched for tags, counted and weighed. Detailed descriptions of sampling methods are in electronic supplementary material, appendix S1.

Because food availability can directly affect instantaneous growth in fishes [33], we sampled benthic macroinvertebrates at each site in April and May 2019. To obtain a measure of available food resource, we used a modified version of the protocol for sampling macroinvertebrates in wadeable streams [34]. Reduced streamflow can increase effective population density and decrease delivery rates of invertebrate drift prey [35]. We therefore recorded current velocity prior to each sampling event using a SonTek FlowTracker (Xylem Analytics, QLD, Australia), and calculated instantaneous stream discharge (m^3^ s^−1^) using the velocity-area method [36]. To account for the availability of terrestrial prey entering streams from the surface, we measured canopy closure at each site. Finally, because instantaneous growth in ectotherms increases with temperature, we recorded hourly stream temperature at each site using HOBO Pendant data loggers (Onset Computer Corporation, MA, USA).

### (c) Model construction and assessment

To assess the relative impacts of DD and DI processes, we followed a similar approach to [14] constructing a candidate set of *a priori* linear mixed effects models capable of explaining variation in fish growth rates. A specific growth rate (SGR, % day^−1^) was used as the response variable for each recaptured fish. Specific growth rates were calculated using

2.1 $$\mathrm{SGR}\;=\;\frac{\log({\mathrm{TL}}_{r})-\log({\mathrm{TL}}_{c})}{\Delta \mathrm{days}}\times 100,$$

where TL_r_ is TL at the time of recapture, TL_c_ is TL at the time of capture and Δdays is time at liberty.

Stream temperature was used as a DI factor because it is known to interact with population density to affect fish growth in natural populations [37]. The mean daily stream temperature (Temp) for each individual fish (*i*) was calculated using

2.2 $${\mathrm{Temp}}_{i}\;=\;\frac{\sum_{c=1}^{r}{\mathrm{temperature}}_{\mathrm{ic}}}{\Delta {\mathrm{days}}_{i}},$$

where temperature is the sum of daily temperatures for each stream between capture (*c*) and recapture (*r*) for an individual fish (*i*), and Δdays is time at liberty.

Stream discharge (m^3^ s^−1^) was treated as a DI factor because reduced current velocities could negatively affect individual *G. maculatus* growth. The mean stream discharge for each individual fish (*i*) between capture (*c*) and recapture (*r*) for each stream was calculated using:

2.3 $$\begin{array}{l} \text{mean stream }{\text{discharge}}_{i}\;\\ \;=\;\frac{\sum_{c=1}^{r}\text{stream }{\text{discharge}}_{ic}}{\text{no}.\text{ sampling events between }c\text{ and }r}. \end{array}$$

To examine the effects of compensatory DD growth affecting *G. maculatus*, a mean population density for each individual fish (*i*) was calculated based on population density for all sampling events within each year between capture (*c*) and recapture (*r*) for each stream using:

2.4 $$\mathrm{population}\;\mathrm{density}=\frac{\sum_{i=1}^{n}G.\;maculatus_{i}}{\mathrm{stream}\;\mathrm{area}}$$

and

2.5 $$\begin{array}{l} \text{mean population }{\text{density}}_{i}\;\\ \;=\frac{\sum_{c=1}^{r}\text{population }{\text{density}}_{c}}{\text{no}.\text{ sample events between }c\text{ and }r}, \end{array}$$

where stream area is the site length (150 m) multiplied by the average wetted width measured for each site during baseflow conditions in the austral spring (October–November 2018 and 2019) and autumn (April–May 2019).

Prior to analyses, all variables were checked for normality, and outliers in the response variable (SGR) were removed. Mean population density and benthic biomass were log-transformed to approximate assumptions of parametric statistics. Benthic biomass and percentage canopy closure were excluded as covariates from the candidate set of growth models because they were highly correlated (Pearson's correlation <|0.5|) with mean population density and stream discharge, respectively. Each candidate model included the initial total length of recaptured fish to account for differences in growth due to body size. We also included a random stream intercept to account for stream-specific differences and a random fish intercept to account for capturing the same fish on multiple sampling occasions. Constructed models included additive and interactive effects between DD, DI and body size because all of these mechanisms can affect instantaneous growth dynamics in temperate stream-dwelling fish populations [20,37,38]. All models had an ecologically realistic, *a priori* basis and were not data mined [39].

Akaike's information criterion (AIC), an information theoretic approach [39], was used to determine the best supported models to explain variability in *G. maculatus* SGR. AIC scores were adjusted for small sample size (AIC_c_) to account for the relatively low number of sites [39]. To classify the level of empirical support for models that explained SGR, ΔAIC_c_ (AIC_c_ – minimum AIC_c_) was calculated, where values between 0 and 2 indicate substantial support, 4 and 7 some support, and greater than 10 no support [39]. We also calculated Akaike weights (*w_i_*) to represent the posterior probability that a model is true, given the data and the set of competing models [39]. Only the most parsimonious models that had *w_i_* values greater than 10% of the model with the greatest *w_i_* were considered [39].

## 3. Results

**Figure 2.**
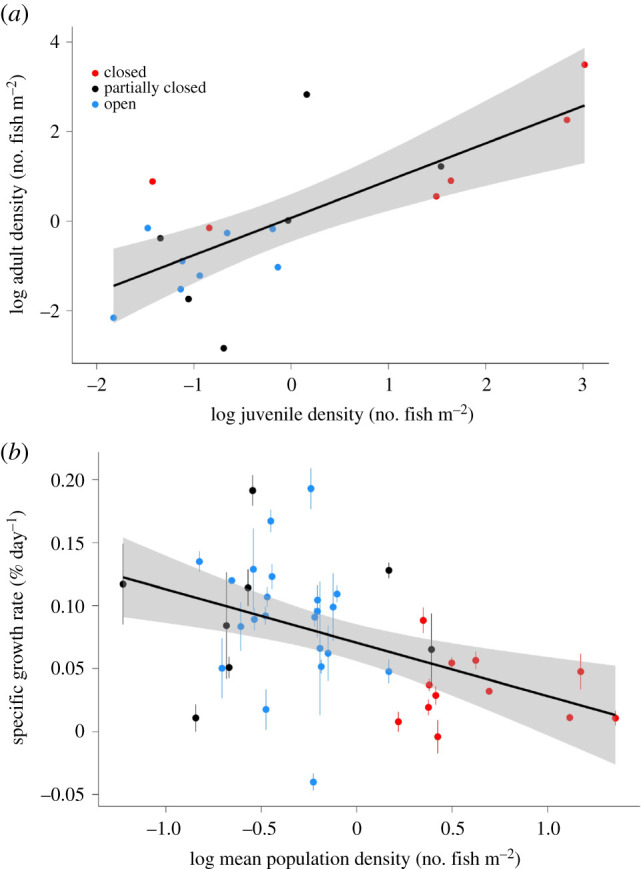
Linear (log-log) relationship between post-recruit (late juvenile and adult) population density (February) and mean recruiting (juvenile) population density (September–December) of īnanga (*Galaxias maculatus*) standardized by stream area (*a*), and the relationship between specific growth rates of post-recruit *G. maculatus* individuals and mean population density of post-recruit populations (January–April) within streams (*b*). (Online version in colour.)

**Figure 3.**
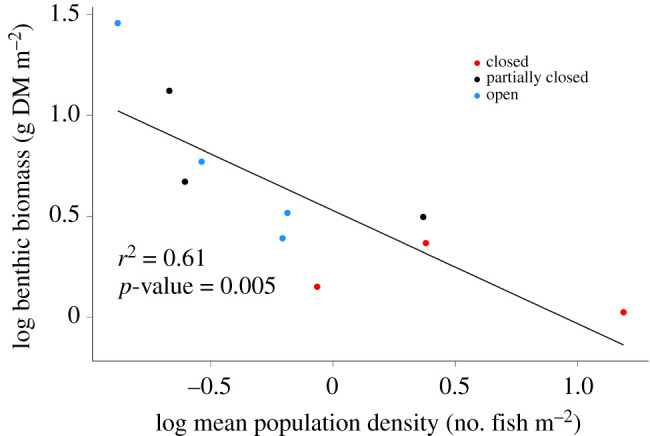
Negative (log-log) correlation between mean population density of *Galaxias maculatus* in 2019 and benthic macroinvertebrate biomass sampled at study sites in April and May of 2019. (Online version in colour.)

Across the 10 populations, regression analysis showed a linear log-log relationship (*r*^2^ = 0.62; *p* < 0.001) between the density of adult fish sampled in February and that of juveniles that recruited during September–December (figure 2*a*). Regression analysis showed a negative relationship (*r*^2^ = 0.23; *p* < 0.001) between specific growth rates of post-recruit fish and the density of populations (figure 2*b*). It is noteworthy that there is considerable variability in growth rates at lower densities of fish (−1.2 to 0.4 on *x*-axis, equating to 0.06–2.5 fish m^−2^), compared to greater densities up to 22.7 fish m^−2^ (greater than 0.5 on *x*-axis). *Galaxias maculatus* population density patterns matched expectations based on stream type, with closed streams (Sites 6, 7 and 9) having greater population densities (range 1.7–22.7 fish m^−2^) than partially closed and open streams (Sites 1, 2, 3, 4, 5, 8 and 10; table 1). The population densities were greatest in Site 7 (closed; 14.9–22.7 fish m^−2^) and lowest in Site 4 (partially closed; 0.1–0.3 fish m^−2^) in both years. Partially closed (0.1–2.5 fish m^−2^) and open streams (0.2–1.5 fish m^−2^) had overlapping population density estimates. Population densities were greater in six of the seven streams sampled in 2019 than in 2020. Stream temperature was greater in 2019 than 2020 in all 10 streams (table 1). Site 4 (partially closed, with the lowest population density) was the warmest and Site 10 (open) the coldest in both years. Stream temperature, used as a predictor in the growth models, was 17.3–20.0°C for closed streams, 14.5–21.2°C for partially closed streams and 13.9–20.3°C for open streams. Stream discharge was greater in 2019 than 2020 at six of the seven sites sampled, and was greatest at Site 3 (open; 0.79–1.06 m^3^ s^−1^) and lowest at Site 6 (closed; 0.01–0.10 m^3^ s^−1^) during both years (table 1). Stream discharge, used as a predictor in the growth models, was 0.01–0.32 m^3^ s^−1^ for closed streams, 0.05–0.64 m^3^ s^−1^ for partially closed streams and 0.08–1.06 m^3^ s^−1^ for open streams. Benthic macroinvertebrate biomass was correlated with density (*r*^2^ = 0.61, *p* = 0.005; figure 3), with greater benthic macroinvertebrate biomass in lower density sites (open and partially closed streams) and lower benthic macroinvertebrate biomass in greater density sites (closed streams). Benthic biomass was 1.06–2.33 g DM m^−2^ for closed streams, 3.14–13.24 g DM m^−2^ for partially closed streams and 2.46–28.63 g DM m^−2^ for open streams.

**Table 1.** Habitat characteristics and īnanga (*Galaxias maculatus*) sampling summaries for each site classified by stream type. Daily temperature (°C, range) is the mean stream temperature from 15 January to 15 April in 2 years. ‘Population density' (no. *G. maculatus* m^−2^) and ‘discharge' (m^3^ s^−1^) are means with standard errors in parentheses. ‘Tagged' is the number of individually marked fish and ‘recaptured' is the number of fish used for growth modelling.

site	stream type	daily temperature		population density		discharge		tagged		recaptured	
		2019	2020	2019	2020	2019	2020	2019	2020	2019	2020
1	partially closed	—	17.1 (11.4–22.4)	—	1.5 (0.5)	—	0.1 (0.0)	—	404	—	53
2	open	18.0 (12.4–21.4)	17.1 (11.6–21.6)	0.6 (0.1)	0.6 (0.2)	0.6 (0.0)	0.4 (0.1)	389	311	91	25
3	open	16.1 (11.5–18.5)	15.3 (10.7–18.8)	0.3 (0.1)	0.2 (0.0)	0.9 (0.2)	0.8 (0.0)	253	263	57	11
4	partially closed	18.9 (12.4–22.0)	17.7 (11.5–22.4)	0.2 (0.1)	0.1 (0.0)	0.5 (0.1)	0.3 (0.0)	409	65	46	3
5	open	16.7 (11.4–21.2)	16.4 (10.8–21.5)	0.6 (0.1)	0.3 (0.0)	0.1 (0.1)	0.1 (0.1)	382	208	88	79
6	closed	17.8 (12.4–23.3)	17.5 (11.2–22.1)	2.4 (0.5)	4.2 (2.0)	0.1 (0.1)	0.0 (0.0)	303	380	125	25
7	closed	16.7 (11.2–21.6)	16.6 (9.3–21.9)	22.7 (10.2)	14.9 (5.3)	0.1 (0.0)	0.2 (0.0)	163	449	14	17
8	partially closed	—	15.9 (10.4–19.7)	—	2.5 (1.8)	—	0.4 (0.1)	—	361	—	3
9	closed	—	16.2 (10.1–19.9)	—	13.1 (10.6)	—	0.3 (0.0)	—	207	—	54
10	open	13.7 (10.3–16.2)	13.5 (9.7–15.8)	0.2 (0.0)	1.5 (0.6)	0.4 (0.2)	0.3 (0.0)	164	259	5	21

### (a) Growth analysis

Of the 4970 tagged fish, there were 717 recaptures between 2019 and 2020 used for analyses (table 1). The greatest number of recaptured fish were from an open stream, Site 5 (*n* = 167) with the fewest from a partially closed stream, Site 8 (*n* = 3). Model selection showed an interaction among initial length, mean daily stream temperature, and mean population density as the most parsimonious model explaining SGR (table 2). This model ranked highest and was 9.87 times more likely to be the best explanation of specific growth rate than the next model, as indicated by Akaike weights (0.908/0.092). The second-ranked model did not include size effects (i.e. no interaction with initial length; table 2). The parameter estimates of both models did not include zero (electronic supplementary material, table S2). Random intercepts from the most parsimonious model indicated that more variability in SGR was explained by stream-specific differences (0.14% ± 0.04 s.d.) than by individuals that were captured more than once (0.06% ± 0.03 s.d.).

**Table 2.** Summary of AIC_c_ model selection and Akaike weights (*w*) for specific growth rates of īnanga (*Galaxias maculatus*) from the West Coast of New Zealand. ‘Model' represents model structure with the parameters included and the *a priori* hypothesis for why that model was constructed. Model structure is represented by initial body size (length, mm), mean daily temperature (temp, °C), mean population density (density, *G. maculatus* m^−2^), and mean stream discharge (*Q*, m^3^ s^−1^) between capture and recapture for individual fish.

model	hypothesis	AIC_c_	ΔAIC_c_	*w*	$R_{m}^{2}$	$R_{c}^{2}$
length × temp × density	similar to length + temp × density hypothesis (below) but with size effects	−2427.7	0.0	0.908	0.51	0.82
length + temp × density	specific growth rate increases with stream temperature and decreases with population density	−2423.1	4.6	0.092	0.49	0.82
length + temp	specific growth rate increases with stream temperature due to metabolic processes scaling with temperature	−2400.0	27.7	0.000	0.39	0.83
length × temp	similar to length + temp hypothesis (above) but with size effects	−2398.2	29.5	0.000	0.39	0.83
length × *Q* × density	similar to length + *Q* × density hypothesis (below) but with size effects.	−2345.1	82.6	0.000	0.30	0.83
length + *Q*	specific growth rate increases with discharge due to increased availability of invertebrate drift	−2327.5	100.2	0.000	0.28	0.82
length × density	similar to length + density hypothesis (below) but with size effects	−2327.2	100.5	0.000	0.28	0.66
length × *Q*	similar to length + *Q* hypothesis (above) but with size effects	−2325.9	101.8	0.000	0.29	0.82
length + *Q* × density	specific growth rate increases with discharge and decreases with population density	−2323.6	104.1	0.000	0.28	0.81
length + density	specific growth rate decreases with density due to DD effects	−2316.1	111.7	0.000	0.25	0.67
length	only size affects specific growth rate	−2315.7	112.1	0.000	0.23	0.73

**Figure 4.**
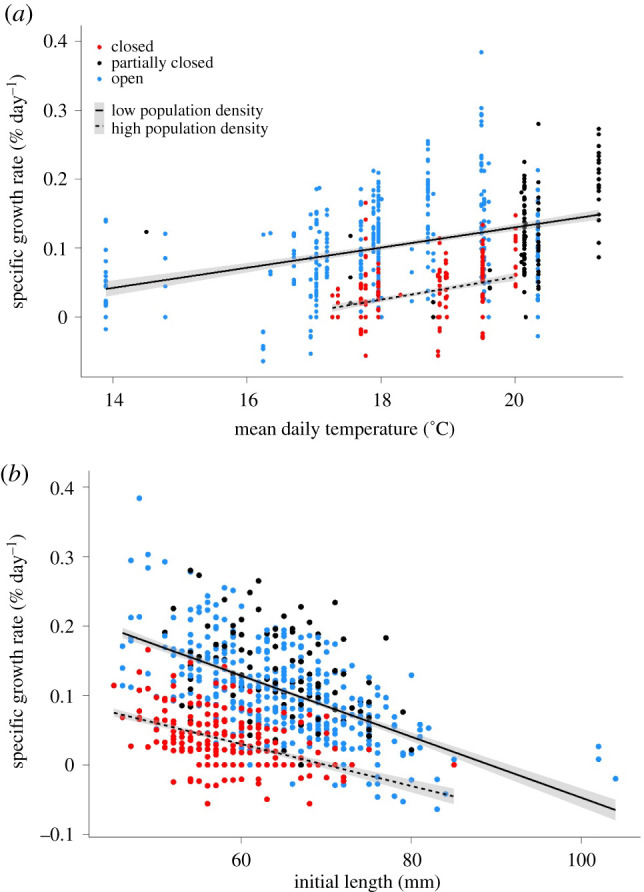
Specific growth rate against mean daily stream temperature (*a*), and specific growth rate against initial total length (*b*) for īnanga (*Galaxias maculatus*) populations on the West Coast of New Zealand. Means are solid black line for low-density (open and partially closed) and dashed black line for high-density (closed) sites. Standard error intervals are represented by grey bands predicted from the most parsimonious model (table 2). (Online version in colour.)

Specific growth rates were positively associated with stream temperature, and negatively associated with mean population density, with a negative temperature:mean population density interaction (electronic supplementary material, table S2). Predicted growth rates were greater in low-density sites (that included open and partially closed streams) and lower in high-density sites (closed streams; figure 4*a*). At a temperature of 20°C, for example, high-density fish are predicted to grow at less than half the rate of low-density fish. To examine size effects on growth, specific growth rates were plotted against initial length. Predicted growth rates from the most parsimonious model (table 2) for large fish were lower than those of small fish in low- and high-density sites (figure 4*b*).

## 4. Discussion

This study presents compelling evidence of interacting effects of population density (density-dependence) and stream temperature (density-independence) on growth rates of post-recruit *Galaxias maculatus* populations. Increased abundance but reduced individual growth rates were observed in fish in FPAs that were implemented to alleviate fishing pressure on *G. maculatus*. This is consistent with other observations [40], although others did not examine interactions between DD and DI effects. By sampling across a range of densities, it was clear that when population densities were high (closed streams) compensatory responses were strongest, indicating that DD growth is a key mechanism regulating post-recruit populations of *G. maculatus*. Additionally, the positive relationship between the density of post-recruits (late juveniles and adults) and recruiting fish is a good indication that juvenile mortality rates in *G. maculatus* are not strongly affected by density. Closed and partially closed streams had the greatest recruitment and therefore should have had the clearest evidence of compensatory mortality if these processes are important [41]. This finding increases the value of FPAs for conservation and fisheries sustainability of *G. maculatus* because juvenile mortalities did not appear to reduce adult populations to a roughly consistent level (i.e. protected and non-protected habitats did not reach an equilibrium adult population level or carrying capacity). The annual life history and shoaling behaviour (described below) of *G. maculatus* may provide explanations as to why DD survival in the juvenile phase appears not to be an important factor in regulating their populations.

We hypothesized that *G. maculatus* growth rates would increase with stream temperature because biochemical reaction rates, metabolic rates, and nearly all other rates of biological activity in ectotherms increase exponentially with temperature until a thermal optimum is reached [15]. Model selection indicated that stream temperature was a significant DI variable affecting growth rates, and the positive relationship between individual growth rate and temperature was consistent with our prediction. Most of the FPAs closed to whitebait fishing were established at low elevations to protect *G. maculatus* [26,30], which are largely confined to lowland coastal watersheds. Watson *et al*. [26] showed that the FPAs included in this study all had similar stream temperatures. Coincidentally, the range of mean daily stream temperature recorded in closed streams during this study was identical to the laboratory determined preferred temperatures of juvenile *G. maculatus* [42]. Adult *G. maculatus* were observed by [42] to select sites for occupation with mean temperatures within 1°C of their preferred temperature (18.1°C) although a significant relationship between population density (fish m^−2^) and temperature was not observed from field records.

All stream temperatures during our study were well below the upper lethal temperature of 30.8°C, and below, but approaching, the predicted optimal growth temperature of 22.5°C for *G. maculatus* [42,43]. However, before our study commenced in the austral summer of 2018/2019, our temperature loggers recorded mean daily stream temperatures above 22.5°C at four of the 10 study sites. Over the previous summer (2017/2018), the New Zealand region experienced a heat wave that covered the whole country as well as the central and south Tasman Sea and across 180° E in the southwest Pacific Ocean [44]. Breaches of physiological temperature thresholds for the kelp *Macrocystis pyrifera*, significant stock mortality of Chinook salmon *Oncorhynchus tshawytscha*, and out-of-range reports of tropical and warm-temperate fish were observed [44]. While [44] only presented data on this unprecedented oceanic-atmospheric heat wave, atmospheric temperatures are also highly correlated with freshwater surface temperatures over large spatial and temporal scales [45]. Similarly to this study, [14] observed declining growth rates in salmonids with warmer temperatures (through a quadratic relationship), indicating that high stream temperatures precluded optimal growth, suggested that the negative consequences of warming trends associated with climate change may be exacerbated by DD effects. Although our study design did not include a temperature gradient across the study streams from which a quadratic relationship between growth and temperature could be determined, our results highlight the need to incorporate climate change predictions in the placement of future FPAs if they are to be used as management tools.

Our prediction that *G. maculatus* growth rates would increase with increasing stream discharge was unsupported. Although [26] showed that stream discharge was lower in closed streams than partially closed and open streams, other studies have shown that increased stream flow results in an increase in drift invertebrates leading indirectly to increased growth [28]. However, it should be noted that our ability to examine such effects was limited because we had only instantaneous measurements of stream flows at study sites, and nearby continuous gauging stations were located on larger rivers that were not suitable for mark-recapture studies. Also, population sampling only occurred during times of low flow because high flow conditions are not suitable for trapping methods.

The prediction that DD effects would more strongly affect smaller individuals, among fish of similar ages, due to mouth gape size limitation was not supported. Instead, the most parsimonious model, that included individual size as an interactive effect with temperature and mean population density, showed that smaller individuals had the greatest growth rates at low and high population densities. Furthermore, the model ranked second did not include an interaction involving initial size. Post-larval *G. maculatus* stay together in shoals, a characteristic that is unique among galaxiids [22]. Shoals are often found cruising large areas, non-aggressively, drift-feeding [46]. The gregarious shoaling and cruising behaviour probably results in an equal share of resources among individuals regardless of their size and provides a good explanation as to why DD effects on growth did not affect smaller individuals more strongly. Similarly [47] found that in lentic environments white-spotted char (*Salvelinus leucomaenis*) showed limited aggression and cruised over large areas in search of food. Cruising behaviour is more consistent with exploitative competition and consequently there was little difference in growth rates among individuals [47].

It is probable that *G. maculatus* growth increases with available forage (i.e. increased terrestrial and stream-based prey). At our study sites, benthic macroinvertebrate biomass decreased as the fish population density increased, and although we did not quantify invertebrate drift, reduced stream flow has been linked to reduced food availability [28]. Additionally, Watson *et al.* [26] showed that large predatory fishes (*Anguilla dieffenbachii* longfin eel, *A. australis* shortfin eel and *G. argenteus* giant kōkopu) were more common in closed streams compared to the partially closed and open streams, which may have exacerbated the limited amount of food available in the dense populations. With less food *per capita*, walleye (*Stizostedion vitreum*) were observed to be unable to maintain growth, which led to delayed maturation and lowered reproductive potential [48]. Like most fishes, *G. maculatus* fecundity increases with body size [49], highlighting the importance of food availability to maintain individual growth.

In addition to DD food availability affecting individual growth rates, food availability just prior to the spawning season may also influence the size and quality of eggs spawned [50]. Egg size has been shown to increase with body size for many species, with larger eggs leading to increased larval survival [51]. Currently, the consequences of DD growth on fecundity and egg size are unknown for *G. maculatus* and this may be a fruitful area for future work. This is especially important for evaluating the overall effectiveness of FPAs, considering that the major subsidy of FPAs for *G. maculatus* is sustainable egg output and ensuring the migration to sea of the greatest possible number of larvae. Estimates of population egg production in smaller adult *G. maculatus* in closed streams have been found to be greater than those in partially closed and open streams [26].

FPAs share many of the ‘reserve effects' of more common MPAs, but there are also important differences [26]. A key contrast is that for FPAs, size matters. MPAs have been associated with increased densities, biomass, individual size, and diversity of fish, regardless of reserve size [7]. When resources in an MPA become limited through increased competition, highly mobile species emigrate in search of more suitable habitats and resources outside of the PA. This net movement of post-settlement individuals from MPAs, or ‘spill-over', is a key benefit for fisheries enhancement derived from MPAs [6]. Adult *G. maculatus* do not migrate between waterways, so the number of juveniles and adults that an FPA can accommodate is ultimately regulated by the extent or availability of habitat [52]. With no adult *G. maculatus* spill-over possible, FPA size becomes a limiting factor, with smaller habitats probably exacerbating compensatory DD processes at high densities. Therefore, even high-quality habitats that produce the most rapid growth and greatest individual fecundity may not be the key to greater egg production, especially if such habitats are sparse in area [30]. Unfortunately, the FPAs considered here do not ensure protection of the surrounding instream and riparian habitat unless located within national parks, and overall, average habitat assessment scores for the closed and partially closed streams in this study were suboptimal [26]. It may be that large areas of mediocre quality habitat are more important to overall, cumulative egg output than small areas of high-quality habitat [30]. Consequently, if FPAs are to be used as effective management tools, the quality and extent of the target-species' habitat are key factors to consider. High-quality and extensive habitats may potentially underpin increases in growth, survival, and fecundity; as well as greater colonisation rates driven by enhanced settlement and recruitment [53]. It is also noteworthy that other species of migratory galaxiids that form a much smaller part of New Zealand's whitebait catch [21] also inhabit the FPAs.

While single-species studies can be useful, especially for fisheries management, it is important to remember that species-specific responses to protection occur [7]. Preliminary research suggests that other migratory galaxiids (*G. fasciatus, G. argenteus* and *G. postvectis*) do not respond to protection in the same manner as *G. maculatus* (B. Crichton 2021, unpublished data). Therefore, for FPAs to succeed, their establishment should be linked with programmes designed to provide information on their effectiveness as management tools, and to direct *a posteriori* adaptive management actions [54]. In principle, once fishing restrictions are in place, and populations are allowed to recover, we should expect opposite compensatory changes in life-history parameters to those described in exploited populations [8]. Indeed, several studies have observed the effect of protection and DD processes, such as growth, on fish populations in MPAs [55], but this study is the first we know of to observe the effect in FPAs.

In conclusion, population density and stream temperature interacted to affect growth rates of post-recruit *G. maculatus*, but juvenile mortality rates were not strongly affected by density. Density-dependent growth links population density to individual size, and because smaller fish produce fewer eggs, this can propagate to the population level. However, this feedback is complicated for *G. maculatus* because it is an annual species and returning post-larvae show no fidelity to natal streams [56]. Nevertheless, the feedbacks between density effects, population egg production and future population structure will be useful for understanding the potential of no-take FPAs to enhance freshwater fisheries. However, it is not just feeding and foraging habitats that need attention, but also requisite riparian spawning habitats. Properly managed FPAs must consider the entire stream-based life-history requirements of this and other species if they are to be more effective. Our results emphasize the importance of considering the effects of DD and DI processes on the function and effectiveness of FPAs as fisheries management tools. Clearly, the consequences of over- or under-estimating DD and DI processes in conjunction with habitats, or ignoring them altogether, in fisheries assessments are serious. Cessation of fishing will not necessarily improve fishery productivity, unless the wider ambit of population processes includes improved habitats.

## Data accessibility

The datasets and underlying code supporting this article are available on figshare: https://doi.org/10.6084/m9.figshare.17072132. The data are provided in electronic supplementary material [57].
